# Indirect Effects of the COVID-19 Pandemic on In-Hospital Outcomes among Internal Medicine Departments: A Double-Center Retrospective Study

**DOI:** 10.3390/jcm12165304

**Published:** 2023-08-15

**Authors:** Maurizio Di Marco, Nicoletta Miano, Simona Marchisello, Giuseppe Coppolino, Giuseppe L’Episcopo, Sabrina Scilletta, Concetta Spichetti, Serena Torre, Roberto Scicali, Luca Zanoli, Agostino Gaudio, Pietro Castellino, Salvatore Piro, Francesco Purrello, Antonino Di Pino

**Affiliations:** Department of Clinical and Experimental Medicine, University of Catania, 95122 Catania, Italy; maurizio.dimarco@studium.unict.it (M.D.M.); nicoletta.miano@gmail.com (N.M.); simomarchi91@hotmail.it (S.M.); giuseppecoppolino93@gmail.com (G.C.); peppe94@gmail.com (G.L.); sabrinascilletta@gmail.com (S.S.); ketty1985@virgilio.it (C.S.); serenatorre@tiscali.it (S.T.); roberto.scicali@unict.it (R.S.); luca.zanoli@unict.it (L.Z.); agostino.gaudio@unict.it (A.G.); pcastell@unict.it (P.C.); salvatore.piro@unict.it (S.P.); francesco.purrello@unict.it (F.P.)

**Keywords:** COVID-19, internal medicine departments, in-hospital outcomes, length of in-hospital stay, in-hospital mortality, sepsis

## Abstract

The coronavirus disease 19 (COVID-19) emergency led to rearrangements of healthcare systems with a significant impact on those internal medicine departments that had not been converted to COVID-19 wards. A reduced number of departments, indeed, had to cope with the same number of patients along with a lack of management of patients’ chronic diseases. We conducted a retrospective study aimed at examiningthe consequences of the COVID-19 pandemic on internal medicine departments that were not directly managing COVID-19 patients. Data from 619 patients were collected: 247 subjects hospitalized in 2019 (pre-COVID-19 era), 178 in 2020 (COVID-19 outbreak era) and 194 in 2021 (COVID-19 ongoing era). We found that in 2020 in-hospital mortality was significantly higher than in 2019 (17.4% vs. 5.3%, *p* = 0.009) as well as length of in-hospital stay (LOS) (12.7 ± 6.8 vs. 11 ± 6.2, *p* = 0.04). Finally, we performed a logistic regression analysis of the major determinants of mortality in the entire study population, which highlighted an association between mortality, being bedridden (β = 1.4, *p* = 0.004), respiratory failure (β = 1.5, *p* = 0.001), glomerular filtration rate (β = −0.16, *p* = 0.03) and hospitalization in the COVID-19 outbreak era (β = 1.6, *p* = 0.005). Our study highlights how the COVID-19 epidemic may have caused an increase in mortality and LOS even in patients not directly suffering from this infection.

## 1. Introduction

The coronavirus disease 19 (COVID-19) pandemic has been an ongoing global emergency since the first case was detected in December 2019; accordingly, from 1 January 2020 to 31 December 2021, a total of 5.94 million COVID-19 deaths were reported worldwide [1] and seroprevalence studies showed that the real prevalence of severe acute respiratory syndrome coronavirus 2 (SARS-CoV-2) infection could be 10 times more than recorded [2,3]. This led many governments around the world to modify population lifestyle through social distancing and lockdown measures to limit the spread of infection. The SARS-CoV-2 pandemic led to several devastating effects not only on directly affected patients, but also on the general population in terms of healthcare, economic and social damage [4]. Regarding mortality, a recent study published in The Lancet demonstrated that the global all-age rate of excess deaths related to COVID-19 was 120.3 deaths per 100,000 people [5]. These data reflect not only deaths due to COVID-19 alone, but also the mortality indirectly caused by the SARS-CoV-2 pandemic. Specifically, the COVID-19 emergency led to rearrangements of healthcare systems including a reduction in outpatient visits, an increased use of telemedicine, a redistribution of healthcare professionals and a different organization of resources in an emergency setting [6,7,8,9]. Among the several arrangements implemented to tackle this burden, hospital wards of almost all medical and surgical specialties were closed or converted into wards for the sub-intensive care of COVID-19 patients, and in many cases entire hospitals were converted into COVID-19 hospitals. Moreover, the number of emergency hospital admissions significantly dropped during the COVID-19 epidemic, with the exception of admissions for falls and fragility fractures that continued at comparable rates [10]. These indirectly COVID-19-related changes were the result of a variety of factors, including societal disruptions, psychological disorders in families of patients severely affected by COVID-19, decreased social interaction, which mitigated the transmission of endemic pathogens, and diminished air pollution [11]. The COVID-19 pandemic had significant indirect effects on the elderly population, including social isolation, malnutrition, and physical inactivity, with a consequent negative impact on their mobility and respiratory function, which provoked a frailty condition [12].

The above-mentioned changes had a significant impact on internal medicine departments that had not been converted to COVID-19 wards. A reduced number of departments had to cope with the same number of patients as before the pandemic and also with a lack of management of patients’ chronic diseases, which had a negative influence on the acute events for which they were admitted. Furthermore, this situation led to increased pressure on internal medicine departments from emergency departments (EDs) due to a lack of beds [13,14].

Based on these considerations, we conducted a retrospective study, which aimed to examine the indirect consequences of the COVID-19 pandemic on internal medicine departments that were not directly managing SARS-CoV-2 patients. In particular, we examined different clinical outcomes during these period to investigate whether COVID-19 influenced the morbidity and mortality of non-SARS-CoV-2 infected patients and whether, after more than one year since the beginning of the pandemic, these data underwent modifications.

## 2. Materials and Methods

### 2.1. Patients and Calculations

We collected clinical-pathological data from the medical records of patients hospitalized in two internal medicine departments of Catania (Azienda Ospedaliera di Alta Specializzazione Garibaldi Nesima, and Azienda Ospedaliera Universitaria—Policlinico/San Marco Gaspare Rodolico) from September to November in the years 2019, 2020 and 2021. These data were collected to obtain a retrospective evaluation of the clinical characteristics of patients hospitalized in internal medicine departments in the pre-COVID-19 (2019), COVID-19 outbreak (2020), and COVID-19 ongoing (2021) eras. According to hospital protocol, all the patients underwent a double negative PCR swab test for SARS-CoV-2 before admission to exclude the possibility of SARS-CoV-2 infection. Moreover, in patients with radiological or clinical elements suspected to be COVID-19, research using a PCR to detect SARS-CoV-2 from a sample of bronchus alveolar lavage was performed.

The data included: (1) age, gender, comorbidities (hypertension, diabetes mellitus, chronic heart failure, chronic kidney disease, previous myocardial infarction, previous stroke, chronic obstructive pulmonary disease [COPD], chronic liver diseases, solid tumors, onco-hematological diseases, and being bedridden), home-therapy drugs; (2) the department from which the patient was transferred to the internal medicine department; (3) clinical events that occurred during hospitalization (death, length of in-hospital stay [LOS], diagnosis of sepsis, the need for blood transfusion, multidrug-resistant [MDR] germ isolation, and respiratory failure); (3) clinical and biochemical characteristics of the patients on admission to internal medicine departments (systolic, diastolic and mean blood pressure [SBP, DBP and MBP], fasting glucose, creatinine, estimated glomerular filtration rate [eGFR], total cholesterol, high-density lipoprotein cholesterol [HDL], low-density lipoprotein cholesterol [LDL], triglycerides, total proteins, albumin, aspartate aminotransferase [AST], alanine aminotransferase [ALT], N-terminal pro-brain natriuretic peptide [NT-proBNP], procalcitonin, high-sensitivity C-reactive protein [hs-CRP], erythrocyte sedimentation rate [ESR], complete blood count, hemoglobin, hematocrit, international normalized ratio [INR], and venous thromboembolism [VTE] risk).

We considered the high/low cut-off of NT-proBNP, procalcitonin, AST, ALT and hs-CRP according to upper laboratory limits as follows: NT-proBNP, 260 pg/mL; procalcitonin, 0.5 µg/L; AST, 34 UI/L; ALT, 55 UI/L; hs-CRP, 0.5 mg/dL.

eGFR was assessed using the chronic kidney disease epidemiology collaboration (CKD-EPI) equation [15]; and VTE risk was estimated using the Padua score [16].

### 2.2. Statistical Analysis

Statistical comparisons of clinical and biomedical parameters were performed using Stat View 6.0 for Windows. Data are given as means SD or median (IQR). Each variable’s distributional characteristics including normality were assessed using the Kolmogorov–Smirnov test. Statistical analysis included ANOVA for continuous variables and the chi-square test for non-continuous variables. A *p*-value less than 0.05 was considered statistically significant. When necessary, numerical variables were logarithmically transformed to reduce skewness, and values are expressed as median and interquartile range.

Furthermore, a logistic regression analysis using a stepwise approach was performed to investigate the association of clinical and biochemical variables with in-hospital mortality. This analysis was performed for each group (pre-COVID-19 era, COVID-19 outbreak era and post-COVID-19 era) separately and also considering the entire population.

### 2.3. Ethics

This retrospective study was approved by the Ethical Board of Catania 2 (N° prot. 370/2021). All procedures performed in studies involving human participants were in accordance with the ethical standards of institutional and/or national research committees and with the Declaration of Helsinki. The requirement for informed consent was waived because of the retrospective design of the data collection.

## 3. Results

Data from 619 patients were collected: 247 subjects were hospitalized in 2019 (pre-COVID-19 era), 178 in 2020 (COVID-19 outbreak era) and 194 in 2021 (COVID-19 ongoing era).

The clinical characteristics of the subjects are presented in Table 1 and the biochemical parameters at admission to the internal medicine departments are shown in Table 2.

### 3.1. Comparison between COVID-19 Outbreak Era, Pre-COVID-19 Era, and COVID-19 Ongoing Era

No differences were found regarding age, number of bedridden patients, number of home-therapy drugs, SBP, DBP or MBP between the COVID-19 outbreak era (2020) and the pre-COVID-19 era (2019) patients. In the COVID-19 outbreak era, patients were mainly admitted from the ED short stay unit (SSU) (58.4%), whereas in 2019, patients were admitted directly from the ED (60.7%). Furthermore, the number of elective admissions was significantly lower in 2020 than in 2019 (1.7% vs. 9.3%, *p* = 0.001) and the number of patients transferred from other departments was significantly higher (19.7% in 2020 vs. 0.8% in 2019, *p* < 0.001). The percentage of subjects with chronic kidney disease (CKD) was significantly higher in 2020 than in 2019 (26.4% vs. 13.4% respectively; *p* < 0.001) and the Padua score was higher in 2020 than in 2019 (3.74 ± 1.95 vs. 3.14 ± 1.8 respectively, *p* = 0.006) (Table 1).

In the COVID-19 outbreak era, patients showed a significantly lower eGFR than in the pre-COVID-19 era (69.8 ± 34.6 vs. 82.7 ± 31.2 mL/min/1.63 m^2^, *p* = 0.001) and INR was higher without reaching statistical significance (1.3 ± 0.5 vs. 1.2 ± 0.33, *p* = 0.12) (Table 2).

In 2020, in-hospital mortality was significantly higher (17.4% vs. 5.3%, *p* = 0.009). In particular, the major cause of death in 2020 was sepsis (47%). Moreover, the LOS was higher in 2020 than in 2019 (respectively, 12.7 ± 6.8 vs. 11.0 ± 6.2, *p* = 0.04), as well as the number of patients who underwent blood transfusion (respectively, 24.7% vs. 16.6%, *p* = 0.04) and the number of sepsis diagnoses (respectively, 27.5% vs. 19.4%, *p* = 0.04). On the contrary, no differences regarding multidrug-resistant germ isolations were found (Table 3).

In the comparison between the COVID 19 ongoing era (2021) and the COVID-19 outbreak (2020) and pre-COVID 19 eras (2019), we did not find differences regarding age, bedridden patients, or the number of home-therapy drugs. As regards sex and VTE risk data, 2021 was closer to 2020 (42.3% of female patients and a Padua score of 3.53 ± 1.63), but SBP was significantly higher in the post-COVID-19 era than in the pre-COVID-19 era (128.3 ± 20.1 vs. 122.9 ±17.1 mmHg, *p* = 0.03) as well as MBP (92.5 ± 12.6 vs. 87.1 ± 13.4 mmHg, *p* = 0.002). The percentage of patients transferred from the ED SSU was significantly higher in 2021 than in 2019 (73.7% vs. 29.1%, *p* < 0.001) and the number of elective admissions was lower (0% vs. 9.3%, *p* < 0.001) (Table 1).

In 2021, eGFR and INR showed values closer to the pre-COVID-19 era (eGFR 75.7 ± 33.5 vs. 82.7 ± 31.2, INR 1.27 ± 0.4 vs. 1.2 ± 0.33). Furthermore, in 2021, the number of patients with NT-proBNP > 260 pg/mL was significantly higher than in 2019 (35.0% vs. 14.2%, *p* < 0.001) (Table 2).

Moreover, in 2021, we observed a significant reduction in in-hospital mortality and blood transfusions in comparison to the COVID-19 outbreak era (respectively, 7.2% vs. 17.4%, *p* = 0.003 and 16.0% vs. 24.7%, *p* = 0.04), as well as a significant increase in home discharges (73.2% vs. 61.2%, *p* = 0.01). However, sepsis was the first cause of death in 2021 (85.7%), with an increase in multidrug-resistant germ isolation (23.2% 2021 vs. 13.4% 2019, *p* = 0.007). As regards LOS, there was a decrease in comparison to the COVID-19 outbreak era; however, values did not reach statistical significance and values were closer to those in 2019 (Table 3).

### 3.2. Analysis of Major Determinants of In-Hospital Mortality in the Study Groups

Subsequently, we performed a logistic regression analysis, which highlighted an association between in-hospital mortality, solid tumors (β = 4.5, *p* = 0.002) and eGFR (β = −0.094, *p* = 0.003) in 2019. In 2020, in-hospital mortality was associated with solid tumors (β = 2.98, *p* = 0.005) and respiratory failure (β = 3.23, *p* = 0.001). In 2021, we highlighted an association between in-hospital mortality, respiratory failure (β = 3.1, *p* = 0.01) and serum albumin (β = −2.8, *p* = 0.01) (Table 4).

Finally, when evaluating the entire study population (Table 5), we found an association between in-hospital death, being bedridden (β = 1.4, *p* = 0.004), respiratory failure (β = 1.5, *p* = 0.001), eGFR (β = −0.16, *p* = 0.03) and hospitalization in the COVID-19 outbreak era (β = 1.6, *p* = 0.005).

## 4. Discussion

In this study, we aimed to investigate the effects of the COVID-19 pandemic on internal medicine departments not directly involved in the management of patients suffering from SARS-CoV-2 infection. The principal findings were: (1) mortality and morbidity increased among patients hospitalized in the COVID-19 era (2020), with a reverse trend the year after the pandemic outbreak; (2) an increased number of diagnoses of sepsis and increased MDR germ isolation in 2020 and 2021; (3) the length of in-hospital stay was significantly longer during 2020. All these results are in line with other studies showing an increase in mortality in patients admitted to the ED and other specialty units during the pandemic [17,18,19,20,21,22,23]. These findings could be explained by a large decrease in hospitalizations during the pandemic due to patients’ fear of hospital-acquired infection of COVID-19, especially among the elderly, and the reduced capacity of healthcare services to provide timely diagnoses and treatment. Indeed, during this period, the percentage of patients with a diagnosis of acute coronary syndrome decreased by 40% compared to the same period in 2019 [24], and hospitalizations for acute stroke decreased by 80% to 50% in some countries [25]. Furthermore, the number of newly diagnosed cancer patients decreased by 46.5% [26]. As regards hospitalizations, the outpatient volume for all diseases decreased by approximately 60% across the United States [27]. The number of outpatient visits for bronchitis decreased by 76.79%, pneumonia by 71.03%, and acute upper respiratory infection by 56.87% in 2020, compared to the same data observed in 2019 [28]. In addition, Piccolo et al. found that in Campania, Italy, the COVID-19 outbreak was linked to a reduction in percutaneous coronary intervention, and the authors speculate that a possible explanation could be the underestimation of chest pain by patients due to a fear of contracting SARS-CoV-2 infection if they had been to hospital [29]. Furthermore, access to telemedicine was difficult for elderly patients, who often have lower IT skills [30]. Consequently, a delay in seeking medical care led to more critically ill patients being admitted during the pandemic and worse in-hospital outcomes. For instance, our study showed that patients hospitalized during the pandemic had worse kidney function, which is an independent predictor of all-cause mortality [31,32,33]. In addition, patients hospitalized in 2020 more frequently had an altered coagulation state, with an increase in INR and higher Padua score, which are both associated with higher in-hospital morbidity and mortality, as previously reported [34,35,36,37]. Moreover, among our patients, a significantly higher incidence of sepsis and chronic renal failure emerged, and sepsis was associated with an increase in multidrug-resistant germ infections [38,39]. In agreement with the literature, our study confirms a strong association between in-hospital mortality, eGFR, being bedridden and the presence of respiratory failure in the logistic regression analysis. Given the strong association between mortality and respiratory failure, an increase in mortality due to unrecognized SARS-CoV-2 infections could be hypothesized; however, all patients examined had a double negative SARS-CoV-2 nasopharyngeal molecular swab at the time of admission to the internal medicine departments and in patients still suspected of having COVID-19 for radiological and clinical reasons, research using a PCR for SARS-CoV-2 on a sample from bronchus alveolar lavage was performed, according to hospital protocols (see Materials and Methods section). Thus, this hypothesis appears unlikely. Analyzing the causes of death, it is interesting to note that the main cause of in-hospital mortality in the COVID-19 outbreak era was sepsis (47% of the causes of death).

Our study demonstrated an increase in LOS in internal medicine departments in the COVID-19 outbreak era. These data are in disagreement with the findings of studies conducted in ultra-specialized departments such as cardiology [20], neurology, and oncology [22]. In these studies, a shorter LOS was found, assuming a more effective management of patients thanks to a reduction in non-urgent services. However, internal medicine departments mainly manage ‘urgent hospitalizations’ and not ‘elective hospitalizations’, thus they hardly benefit from a reduction in outpatient activities. On the contrary, internal medicine patients are complex, suffering from multiple comorbidities and often malnourished [40]. The management of these patients failed due to a reduction in chronic disease assistance during the COVID-19 outbreak era. This fact has a negative impact on internal medicine specialists who often have to deal with both acute and chronic diseases with their home therapies, which, whether properly managed or not, lead to early hospital readmission. Indeed, our study found a higher prevalence of congestive heart failure and higher blood pressure values among hospitalized patients during 2021 than in previous years.

In light of the differences reported between the three years considered in terms of hospital outcomes, we do believe that some strategies could be embraced in order to reduce healthcare-associated infections and avoid worse outcomes. We suggest using web-based tools and learning from the scientific literature so that member hospitals can compare their performance to the best performers and identify strategies for improvement. Moreover, we recommend having multidisciplinary teams in order to ensure better medical management. We should consider telemedicine to enhance the quality of medical services and to coordinate emergency systems. Remote patient monitoring with the transmission of physiological data from a home setting, could be particularly useful for chronic conditions such as diabetes or hypertension [41]. Telehealth has a lot of advantages, especially during an infectious disease outbreak. However, it requires a change in medical training across all specialties [42]. Eventually, given that the leading cause of death in internal medicine departments is sepsis, we could create our own sepsis protocol for identifying patients at risk. This could allow clinicians to identify and treat patients early. This study has some strengths. To the best of our knowledge, this is the first study to evaluate the impact of the COVID-19 pandemic on clinical outcomes in a specific clinical setting such as internal medicine departments not directly involved in the pandemic emergency. Our findings provide a new perspective for targeting vulnerable patients and helping to make healthcare decisions after the epidemic subsides or before the next wave hits. Lastly, this study is one of the few that has evaluated the change in LOS over the pandemic period in patients with different medical conditions. However, there are some limitations. Firstly, this is an observational study that might not deduce the causal effects of the COVID-19 pandemic in different waves on in-hospital mortality and LOS. However, because of the small differences in the prevalence of comorbidities among the three years, increased in-hospital mortality and decreased LOS in the year 2020 are likely attributable to the COVID-19 pandemic. Secondly, our data could show some heterogeneity due to dicentric collection.

## 5. Conclusions

In conclusion, our study highlights how the COVID-19 pandemic caused an increase in mortality, incidence of sepsis and LOS even in patients not directly suffering from SARS-CoV-2 infection. However, it seems reassuring to note that, just over a year after the onset of the pandemic, the data regarding in-hospital mortality, renal function and coagulation state showed a clear reverse trend. On the other hand, the data concerning an increased prevalence and worse control of chronic diseases at a territorial level such as congestive heart failure and hypertension should be considered as a warning sign. Thus, it could be advisable to have a multidisciplinary approach integrated with telemedicine to ensure better medical management of chronic diseases and avoid worse in-hospital outcomes.

## Figures and Tables

**Table 1:** Clinical characteristics of the study population.

	Pre-COVID-19 Era (2019) (*n* = 247)	COVID-19 Outbreak Era (2020) (*n* = 178)	COVID-19 Ongoing Era (2021) (*n* = 194)
Age—years	65 ± 18.3	67.6 ± 17.8	68.6 ± 16.2
Sex—female *n*. (%)	120 (48.6%)	58 (32.6%) *	82 (42.3%)
DEPARTMENT OF PROVENANCE—*n*. (%)			
Emergency department	150 (60.7%)	36 (20.2%) *	35 (18.2%) *
ED SSU	72 (29.1%)	104 (58.4%) *	143 (73.7%) *
Elective admissions	23 (9.3%)	3 (1.7%) *	0 (0%) *
Transferred from other departments	2 (0.8%)	35 (19.7%) *	16 (8.1%)
Bedridden subjects—*n*. (%)	67 (27.3%)	65 (36.5%)	67 (34.5%)
COMORBIDITIES—*n*. (%)			
Hypertension	138 (55.9%)	107 (60.1%)	114 (58.8%)
Myocardial infarction history	38 (15.4%)	27 (15.2%)	30 (15.4%)
Chronic heart failure	48 (19.4%)	31 (17.4%)	68 (35.1%) * †
COPD	56 (22.7%)	37 (20.8%)	41 (21.1%)
Chronic liver diseases	28 (11.3%)	27 (15.1%)	22 (11.3%)
Diabetes mellitus	86 (34.8%)	53 (29.8%)	57 (29.4%)
CKD	33 (13.4%)	47 (26.4%) *	43 (22.2%)
Solid tumors	67 (27.1%)	47 (26.4%)	43 (22.2%)
Onco-hematological diseases	28 (11.3%)	16 (9.0%)	21 (10.8%)
Number of drugs in home-therapy	5.7 ± 4	5.7 ± 4	5.8 ± 4
VTE risk (Padua Score)	3.14 ± 1.8	3.74 ± 1.95 *	3.53 ± 1.63
SBP—mmHg	122.9 ± 17.1	127.0 ± 21.3	128.3 ± 20.1 *
DBP—mmHg	70.1 ± 10.6	71.1 ± 10.9	74.7 ± 11.1 * †
MBP—mmHg	87.1 ± 13.4	89.9 ± 13.2	92.5 ± 12.6 *

Data are presented as percentage or mean ± SD. ED SSU: emergency department short stay unit; COPD: chronic obstructive pulmonary disease; CKD: chronic kidney disease; VTE: venous thromboembolism; SBP: systolic blood pressure; DBP: diastolic blood pressure; MBP: mean blood pressure. * *p* < 0.05 vs. 2019; † *p* < 0.05 vs. 2020.

**Table 2:** Blood test parameters at admission to internal medicine departments.

	Pre-COVID-19 Era (2019) (*n =* 247)	COVID-19 Outbreak Era (2020) (*n* = 178)	COVID-19 Ongoing Era (2021) (*n* = 194)
Fasting glucose—mg/dL	110.14 ± 47	114.87 ± 53.9	102.35 ± 53.4
Urea—mg/dL	47.83 ± 32.9	63 ± 53.5 *	49.73 ± 40.6 †
Creatinine—mg/dL	1 ± 1.05	1.43 ± 1.56 *	1.35 ± 1.78
eGFR—mL/min/1.73 m^2^	82.7 ± 31.2	69.8 ± 34.6 *	75.7 ± 33.5
Albumin—g/dL	3.15 ± 0.6	3.07 ± 0.6	3.4 ± 2.9
Total bilirubin—mg/dL	1.29 ± 2.14	1.26 ± 2.02	1.92 ± 4.02
AST > 34 UI/L—*n*. (%)	68 (27.5%)	52 (29.2%)	64 (33.0%)
ALT > 55 UI/L—*n*. (%)	30 (12.1%)	30 (16.8%)	41 (21.1%)
NT-proBNP > 260 pg/mL—*n*. (%)	35 (14.2%)	39 (21.9%)	68 (35.0%) *
ESR—mm	59.96 ± 34	55.53 ± 31.72	70.97 ± 115.94
hs-CRP > 0.5 mg/dL—*n*. (%)	212 (85.8%)	151 (84.8%)	161 (83.0%)
Procalcitonin > 0.5 µg/L—*n*. (%)	35 (14.2%)	44 (24.7%) *	51 (26.3%) *
HB—g/dL	11.14 ± 3.25	10.94 ± 2.06	10.62 ± 2.29
INR	1.2 ± 0.33	1.3 ± 0.5 *	1.27 ± 0.4

Data are presented as percentage or mean ± SD. eGFR: estimated glomerular filtration rate; AST: aspartate aminotransferase; ALT: alanine aminotransferase; NT-proBNP: N-terminal pro-brain natriuretic peptide; ESR: erythrocyte sedimentation rate; hs-CRP: high-sensitivity C-reactive protein; HB: hemoglobin; INR: international normalized ratio. * *p* < 0.05 vs. 2019; † *p* < 0.05 vs. 2020.

**Table 3:** In-hospital outcomes.

	Pre-COVID-19 Era (2019) (*n =* 247)	COVID-19 Outbreak Era (2020) (*n* = 178)	COVID-19 Ongoing Era (2021) (*n* = 194)
TYPE OF DISCHARGE—*n*. (%)			
Home discharge	196 (79.3%)	109 (61.2%) *	142 (73.2%) †
Nursing home discharge	16 (6.5%)	16 (9.0%)	16 (8.2%)
Death	13 (5.3%)	31 (17.4%) *	14 (7.2%) * †
Transfer to another department	22 (8.9%)	21 (11.8%)	20 (10.3%)
Voluntary discharge	0 (0%)	1 (0.6%)	2 (1.0%)
Length of in-hospital stay (days)	11 ± 6.2	12.7 ± 6.8 *	11.10 ± 6.64
Blood transfusions—*n*. (%)	41 (16.6%)	44 (24.7%) *	31 (16.0%) †
Diagnosis of sepsis—*n*. (%)	48 (19.4%)	49 (27.5%) *	54 (27.8%) *
MDR germs isolation—*n*. (%)	33 (13.4%)	33 (18.5%)	45 (23.2%) *
Respiratory failure—*n*. (%)	43 (17.4%)	44 (24.7%)	35 (18.0%)

Data are presented as percentage or mean ± SD. MDR: multidrug resistant. * *p* < 0.05 vs. 2019; † *p* < 0.05 vs. 2020.

**Table 4:** Logistic regression analysis evaluating major determinants of in-hospital mortality in the three different eras.

	Coefficient β	*p*
**Pre-COVID-19 era (2019)**		
Solid tumors	4.5	0.002
eGFR	−0.094	0.003
**COVID-19 outbreak era (2020)**		
Solid tumors	2.98	0.005
Respiratory failure	3.23	0.001
**COVID-19 ongoing era (2021)**		
Respiratory failure	3.1	0.01
Serum albumin	−2.8	0.01

eGFR: estimated glomerular filtration rate.

**Table 5:** Logistic regression analysis evaluating major determinants of in-hospital mortality considering the entire population.

	Coefficient β	*p*
Being bedridden	1.4	0.004
Respiratory failure	1.5	0.001
eGFR	−0.16	0.03
Hospitalization in COVID-19 outbreak era (2020)	1.6	0.005

eGFR: estimated glomerular filtration rate.

## Data Availability

Data for this study are available under request addressed to A.D.P.
